# TRPM7 promotes the epithelial–mesenchymal transition in ovarian cancer through the calcium-related PI3K / AKT oncogenic signaling

**DOI:** 10.1186/s13046-019-1061-y

**Published:** 2019-02-28

**Authors:** Lu Liu, Nayiyuan Wu, Ying Wang, Xiaoyun Zhang, Bing Xia, Jie Tang, Jingting Cai, Zitong Zhao, Qianjin Liao, Jing Wang

**Affiliations:** 1 Hunan clinicaI research center in gynecologic cancer, Hunan Cancer Hospital and The Affiliated Cancer Hospital of Xiangya School of Medicine, Central South University, 283, Tongzipo Road, Changsha, 410013 Hunan People’s Republic of China; 2 University of South China, Hengyang, 421001 People’s Republic of China

**Keywords:** TRPM7, EMT, Metastasis, PI3k/AKT, Calcium, Ovarian cancer

## Abstract

**Background:**

The epithelial-mesenchymal transition (EMT) is crucial for metastasis and positively regulated by calcium-related signaling. The melastatin-related transient receptor potential 7 (TRPM7) regulates a non-selective cation channel and promotes cancer metastasis. However, the mechanisms underlying the action of TRPM7 in ovarian cancer are unclear.

**Methods:**

The expression of TRPM7 and EMT markers (Vimentin, N-cadherin, Twist and E-cadherin) in ovarian cancer samples was detected. TRPM7was knockdown by shRNA in Ovarian cancer cell lines to examine calcium [Ca2+]i, EMT markers and PI3K/AKT markers. Various cellular assays, such as invasion and migration, were performed in vitro, and further confirmed in vivo.

**Results:**

TRPM7 expression is negatively correlated with E-cadherin, but positively with N-cadherin, Vimentin and Twist expression in ovarian cancer samples. TRPM7 depletion inhibited the migration and invasion in SKOV3 and OVCAR3 cells. In addition, TRPM7 silencing decreased the lung metastasis of SKOV3 tumors and prolonged the survival of tumor-bearing mice. Similar to that of TRPM7 silencing, treatment with MK886, a potent 5-lipoxygenase inhibitor to reduce TRPM7 expression, and/or BAPTA-AM, an intracellular calcium chelator, significantly mitigated the Epidermal growth factor (EGF) or Insulin-like growth factors (IGF)-stimulated migration, invasion, and the EMT in ovarian cancer cells by decreasing the levels of intracellular calcium [Ca2+]i. Furthermore, treatment with LY2904002, a PI3K inhibitor, also inhibited the migration, invasion, and treatment with both LY2904002 and BAPTA-AM further enhanced their inhibition in ovarian cancer cells. Moreover, treatment with BAPTA-AM mitigated the IGF-stimulated migration, invasion, particularly in TRPM7-silenced ovarian cancer cells. Finally, TRPM7 silencing attenuated the PI3K/AKT activation, which was enhanced by BAPTA-AM, MK886 or LY2904002 treatment in ovarian cancer cells.

**Conclusions:**

TRPM7 silencing inhibited the EMT and metastasis of ovarian cancer by attenuating the calcium-related PI3k/AKT activation. Our findings suggest that TRPM7 may be a therapeutic target for intervention of ovarian cancer.

**Electronic supplementary material:**

The online version of this article (10.1186/s13046-019-1061-y) contains supplementary material, which is available to authorized users.

## Introduction

Ovarian cancer is one of the common malignant tumors in women and its incidence is increasing in the world [1]. Ovarian cancer has unique characters of difficult diagnosis and early metastasis. However, the molecular mechanisms underlying the metastasis of ovarian cancer have not been clarified. Hence, revealing the molecular mechanism of ovarian cancer metastasis will be of significance in management of patients with ovarian cancer.

Tumor metastasis is one of the main biological characteristics of malignant tumors [2]. Previous studies have shown that the epithelial-mesenchymal transformation (EMT) process is an early event of tumor invasion and metastasis [3]. During the process of EMT, epithelial cells lose their polarity and transform into mesenchymal cells, accompanied by up-regulating the expression of N-cadherin, Vimentin, Snail, Slug, Twist and Zeb1/2 as well as matrix metalloproteinase (MMP), but down-regulating the epithelial marker of E-cadherin expression [4, 5]. Although many factors can induce the EMT process in tumor cells [6–9] the precise mechanisms by which regulate the EMT process in ovarian cancer remain unclear.

Recent studies have indicated that the calcium-related signaling, such as the PI3K/AKT pathway, is crucial for the EMT process of cancer [10, 11]. High levels of intracellular calcium [Ca2+]i promote the EMT process and metastasis, and regulate E-cadherin, N-cadherin, Vimentin and α-smooth muscle actin (α-SMA) expression [12]. Furthermore, inhibition of AKT activation decreases the expression of F-actin and Vinculin, two key factors for cytoskeleton and adhesion [13], and attenuates the proliferation and invasion of ovarian cancer cells [14]. However, the key factors to regulate the levels of [Ca2+]i and related PI3K/AKT signaling as well as the EMT process in ovarian cancer have not been clarified.

It is well known that the function of ion channels, such as transient receptor potential melastatin 7 (TRPM7), is one of the important factors to determine the levels of [Ca2+]i [15] and regulates the development, progression and metastasis of cancer [16]. TRPM7 has unique features of cation channels that are highly permeable to divalent cations, including calcium, magnesium and other monovalent cations to increase the levels of [Ca2+]i [17, 18]. Furthermore, TRPM7 acts as a kinase to activate itself and other substrates in an ATP-dependent manner. TRPM7 silencing inhibits the migration of fibroblasts [19]. Our previous study has shown that TRPM7 is highly expressed in ovarian cancer tissues and cells, especially in metastatic ovarian cancer tissues, and the expression levels are positively correlated with pelvic lymph node metastasis and poor prognosis in ovarian cancer patients [20]. Accordingly, we hypothesize that TRPM7 may regulate the EMT process of ovarian cancer cells by regulating the levels of [Ca2+]i to activate the PI3K/AKT signaling, leading to invasion and metastasis of ovarian cancer. In this study we tested the hypothesis in ovarian cancer cells and tumors in vivo.

## Materials and methods

### Tissue samples

A total of 60 patients with ovarian cancer were recruited in the Department of Gynaecology of Cancer Hospital, Xiangya Medical College of Central South University of China. Another 20 patients with non-tumor were also recruited (Additional file 1). All patients did not receive radiotherapy and chemotherapy before surgery. Their surgical ovarian tissues were obtained. Written informed consent was obtained from individual patients. The experimental protocol was carried, according to the ethical standards established by the Declaration of Helsinki and approved by the Joint Ethics Committee of Hunan Cancer Hospital and Affiliated Tumor Hospital of Xiangya Medical College of Central South University of China.

### Immunohistochemistry (IHC)

The specimens were paraffin embedded, and the tissue sections (3 μm) were dewaxed, rehydrated, blocked with 3% BSA and subjected to antigen retrieval. After being washed, the sections were incubated with antibodies against E-cadherin (1: 700, #14472), Vimentin (1: 700, #5741), Twist (1: 700, #46702), Akt (1: 1000, #2920), phospho-Akt (1: 1000, #4060, Cell Signaling Technology, Saint Louis, USA), TRPM7 (1: 700, ab109438), PI3K (1: 1000, ab151549, Abcam) at 4 °C overnight. Mouse IgG was used as negative control. After being washed, the bound antibodies were detected using horseradish peroxidase (HRP)-conjugated goat anti-rabbit IgG (A0208) and goat anti-mouse IgG (A0216, Beyotime, China) and visualized by DAB, followed by counterstaining with hematoxylin. The results were evaluated under a microscope by two pathologists in a blinded manner.

### Cells and culture

Human ovarian cancer CAOV-3, A2780, OVCAR3, PA-1 and SKOV3 cells were obtained from the Cancer Institute, Central South University. CAOV-3, A2780, OVCAR3, PA-1 and SKOV3 cells were cultured in RPMI1640 and DMEM (Gibco, Life Technologies, USA) supplemented with 10% FBS (Zeta Life, France), 100 μg / mL penicillin and 100 U / mL streptomycin at 37 °C in a 5% CO_2_, respectively.

### Establishment of stably TRPM7 silencing cell lines

SKOV3 and OVCAR3 cells (5 × 10^5^/ml) were cultured overnight and transfected with pCMV-Con-sh, pCMV-TRPM7-sh1, pCMV-TRPM7-sh2, or pCMV-TRPM7-sh3 (Shanghai Gene Pharma, China) using Lipofectamine 3000, according to the manufacturer’s instruction (Invitrogen, USA). The target sequences of siRNA1, siRNA2 and siRNA3 were 5’-GGTGTTCCCAGAAAGGCAA-3′, 5’-AACCGGAGGTCAGGTCGAAAT-3′ and 5’-AAGCAGAGTGACCTGGTAGAT-3′, respectively. Two days later, the cells were treated with G418 (500 μg/ml, Biofrox, Germany) for two weeks. The cell clones were examined for TRPM7 silencing by quantitative real-time PCR and western blot.

### Morphology

Lung tissues were obtained from individual nude mice and fixed in Bouin’s solution and 4% formalin, and paraffin-embedded. The lung tissue sections (3 μm) were stained with hematoxylin and eosin (H + E) and examined under a light microscope.

### Immunofluorescent assay

SKOV3-Con-sh, SKOV3-TRPM7-sh, OVCAR3-Con-sh and OVACAR-TRPM7-sh cells were stained with mouse anti-F-actin (ab176753, 1:300, Abcam). After being washed, the cells were stained with Alexa Fluor488-conjugated goat anti-mouse IgG (1:1000, Life Technology, USA) and counterstained with DAPI. In addition, these cells were stained with rabbit anti-E-cadherin (ab15148, 1: 25, Abcam) and mouse anti-Vimentin. After being washed, the cells were stained with Alexa Fluor488-conjugated goat anti-mouse IgG and Alexa Fluor594-conjugated goat anti-rabbit IgG, followed by counterstaining with DAPI. Moreover, the cells were treated with 30 μM MK886 (Sigma) for 72 h and stained with Phalloidin-iFluor 488 Reagent CytoPainter (ab176753, 1:300, Abcam company), followed counterstaining with DAPI (C1005, Beyotime). The cells were examined under a fluorescent microscope.

### Cell migration and invasion assays

SKOV3-Con-sh, SKOV3-TRPM7-sh, OVCAR3-Con-sh and OVCAR3-TRPM7-sh cells (7 × 10^4^/well) were cultured in the upper chamber of 24-well transwell plates (8 μm, Corning, USA) in the presence or absence of 70 ng/ml EGF or 20 μM BAPTA (Sigma). The bottom chambers were filled with complete medium. After culture for 24 h, the cells on the surface of the upper chamber were removed with a cotton swab and the migrated on the bottom surface of the upper chamber were fixed in 4% paraformaldehyde and stained with 0.1% crystal violet. The migrated cells were counted under a microscope in a blinded manner. Similarly, these cells (1.4 × 10^5^/well) were tested for their invasion using Matrigel (BD Biosciences) coated upper chambers. In addition, SKOV3 and OVCAR3 cells were tested for their migration and invasion in the presence or absence of 30 μM MK886 and/or 70 ng/ml EGF, 30 μM MK886 and/or 20 μM BAPTA-AM, 20 μM BAPTA-AM and/or 10–15 μM LY294002 (Sigma), or 20 μM BAPTA-AM and/or 70 ng/ml IGF (Peprotech, New Jersey, USA).

### Wound healing assay

SKOV3, SKOV3-Con-sh, SKOV3-TRPM7-sh, OVCAR3, OVCAR3-Con-sh and OVCAR3-TRPM7-sh cells were cultured in 6-well plates up to 80% confluency. The cells were artificially wounded using a pipette tip and cultured for 48 h in the presence or absence of 30 μM MK886 and/or 70 ng/ml EGF, 30 μM MK886 and/or 20 μM BAPTA-AM, 20 μM BAPTA-AM and/or 10–15 μM LY294002, or 20 μM BAPTA-AM and/or 70 ng/ml IGF. The remaining wounded areas were photographed under an inverted microscope and measured.

### Quantitative real-time PCR (qRT-PCR)

Total RNA was extracted from individual tissues using Trizol reagent, and reversely transcribed into cDNA using Revert Aid First Strand cDNA Synthesis Kit (K1622, Thermoscientific, USA), according to the manufacturer’s instruction. The relative levels of target to the control GAPDH mRNA transcripts were determined by qRT-PCR using the FastStart Essential DNA Green Master kit (06924204001, Lifescience. Roche, Mannheim, Germany) and specific primers in the RocheLightCycler® 96 instrument and software (05815916001, Lifescience). The sequences of primers were.

TRPM7: forward 5′- tcctcaaatcagggcatctt-3′ and reverse 5′- tcttccacagcaaaccactg-3′;

Vimentin: forward 5’-GAAGAGAACTTTGCCGTTG-3′, and reverse 5’-TCCAGCAGCTTCCTGTAGGT-3′; E-cadherin: forward 5’-AGGAATCCAAAGCCTCAGGT-3′ and reverse.

5’-ACCCACCTCTAAGGCCATCT-3′; GAPDH: forward 5’-GAAGGTGAAGGTCGGAGTC-3′ and reverse 5’-GAAGATGGTGATGGGATTTC-3′. The PCR reactions were performed in triplicate and the data were analyzed by 2^-ΔΔCt^.

### Western blot

Ovarian cancer cells were treated with vehicle or MK886, EGF, LY294002, IGF, BAPTA-AM as described doses and the relative levels of target protein expression were determined by Western blot. Briefly, cell lysates (30–50 μg/lane) were separated by sodium dodecyl sulfate-polyacrylamide gel electrophoresis (SDS-PAGE) on 10% gels and transferred to polyvinylidene difluoride (PVDF) membranes. The membranes were blocked with 5% bovine serum albumin (SIGMA, USA) in TBST and incubated overnight at 4 °C with specific antibodies against E-cadherin, Vimentin, Twist, Akt phosphor-Akt, TRPM7, PI3K, and GAPDH. After being washed, the bound antibodies were detected with HRP-conjugated second antibodies and visualized with the ECL Western blotting substrate (32,109, ThermoScientific, USA). The relative levels of individual proteins to control GAPDH were determined by densitometric analysis using the ImageJ software (Madison, WI, USA).

### Measurement of intracellular-free Ca2+ [Ca2+]i

Ovarian cancer cells were treated with MK886 or BAPTA-AM for 24 or 12 h, respectively. The control cells were treated with calcium-free or calcium containing Hank’s balanced salt solution (HBSS, Invitrogen). The cells were washed with HBSS; and pre-incubated with 5 μM Fluo 8-AM (ab142773, Abcam) at 37 °C for 60 min. After being washed, the cells were cultured in calcium-free or calcium containing HBSS at 37 °C for 30 min. Fluorescent signals were captured by a confocal laser scanning fluorescence microscope (LSM 700; Zeiss, Oberkochen, Germany). The data were analyzed using the ZEN image software. Some cells were harvested and re-suspended with Ca^2+^-free HBSS. The contents of [Ca^2+^]i in individual groups of cells were analyzed by flow cytometry.

### Animal experiments

Female BALB/c nude mice at 6 weeks of age were from SLA Laboratory Animal (Changsha, China) and housed in a specific pathogen-free facility. Individual mice were injected intravenously with 5 × 10^6^ SKOV3-Con-sh or SKOV3-TRPM7-sh cells. Four weeks after inoculation, the mice were sacrificed and their lung tissues were dissected out, weighed and photographed. The lung tissues were fixed in 10% formalin and paraffin-embedded. The lung tissue sections (3 μm) were stained with H&E and the metastatic nodules in the lungs were counted in blinded manner. In addition, other individual mice received intravenously the same numbers of cells and the survival of individual mice was monitored up to death. The experimental protocol was approved by the Animal Care Committee of Hunan Tumor Hospital and the Affiliated Tumor Hospital of Xiangya Medical College (Changsha, China).

### Statistical analysis

Data are expressed as the mean ± SEM. The difference among groups was determined by ANOVA and post hoc *Bonferroni* analysis and the difference between groups was analyzed by Student’s T-test. The survival of animals was estimated using the Kaplan–Meier method and analysed by log-rank test. The relationship between two measures was analysed by Spearman’s rank test. The potential risk of individual measures for metastasis was determined by univariate and multivariate analysis using Cox regression model after adjusting for baseline characteristics. All statistical analyses were performed using the SPSS version 15.0 (SPSS, Chicago, IL, USA). A *P*-value of < 0.05 was considered statistically significant.

## Results

### TRPM7 is closely related to the EMT process in ovarian cancer

**Fig. 1:**
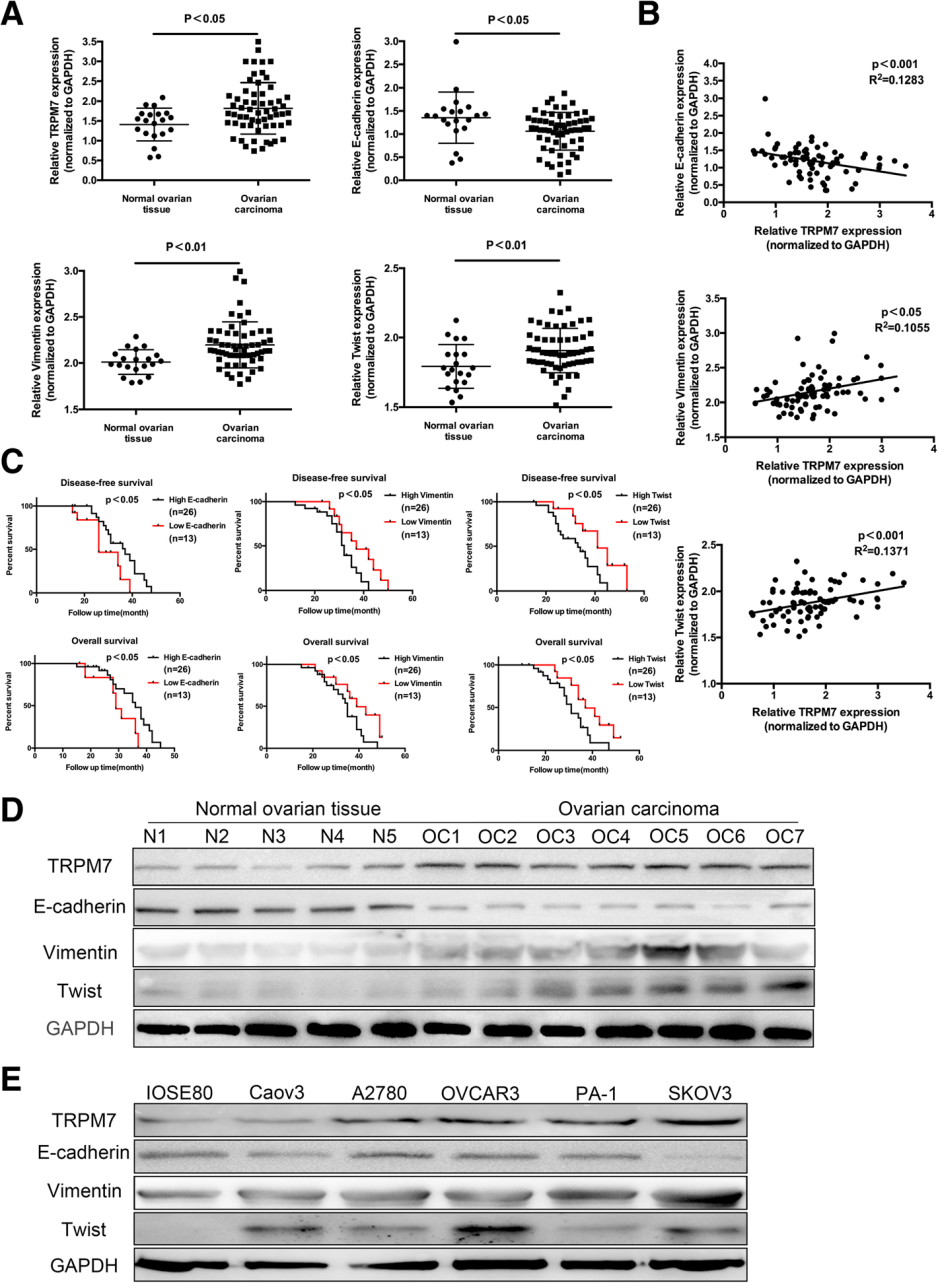
Up-regulated TRPM7 is associated positively with EMT in ovarian cancer. (**a**) The relative levels of TRPM7, E-cadherin, vimentin and Twist mRNA transcripts in 20 non-tumor ovarian and 60 distant metastatic ovarian cancer tissues were examined by qRT-PCR. (**b**) Correlation between TRPM7 and E-cadherin, vimentin or Twist mRNA transcripts was analyzed. (**c**) Western blot analysis the relative levels of TRPM7, E-cadherin, vimentin and Twist expression in 20 non-tumor ovarian and 60 distant metastatic ovarian cancer tissues. (**d**) The disease-free and overall survival in ovarian cancer patients. The patients were stratified, according to the mean value of each measure in this population. (**e**) Western blot analysis of the levels of TRPM7, E-cadherin, vimentin and Twist expression in ovarian cancer cells

The EMT process is crucial for distant metastasis of cancer. To understand the potential role of TRPM7 in promoting metastasis, the relative levels of TRPM7, E-cadherin, Vimentin, and Twist to the control mRNA transcripts in 60 ovarian cancer and 20 non-tumor ovarian tissues were determined by quantitative RT-PCR. The relative levels of E-cadherin mRNA transcripts in ovarian cancer tissues were significantly lower than that in the non-tumor tissues (*p* = 0.0132) while the relative levels of TRPM7 (*p* = 0.0104), Vimentin (*p* = 0.0021) and Twist (*p* = 0.0068) were significantly higher than that in the non-tumor tissues in this population (Fig. 1a). Further analyses indicated that the levels of TRPM7 expression were negatively correlated with E-cadherin (*p* = 0.0017), but positively with Vimentin ((*p* = 0.0107), and Twist (*p* = 0.0007) in ovarian cancer tissues (Fig. 1b). Western blot analysis revealed a similar pattern of TRPM7, E-cadherin, Vimentin and Twist expression in some ovarian cancer and non-tumor tissues (Fig. 1c). Stratification analysis indicated that higher levels of E-cadherin, but lower TRPM7, Vimentin and Twist expression were associated significantly with longer disease-free and overall survival in this population (Fig. 1d). Multivariate analysis revealed that high levels of Vimentin, Twist expression and lower levels of E-cadherin expression were independent risk factors for poor prognosis of patients with ovarian cancer (Table 1). Finally, similar patterns of TRPM7, E-cadherin, Vimentin and Twist expression were observed in ovarian cancer cells and non-tumor ovarian epithelial cells (Fig. 1e). Given that lower E-cadherin but higher Vimentin and Twist expression are hallmarks of EMT process in cancer our data indicated that up-regulated TRPM7 expression was associated with the EMT process of ovarian cancer.

**Table 1:** Mutivariate Analyses for Part Patients (*n* = 39)

Characteristics	DFS		OS	
	HR(95%CI)	*P*-value	HR (95%CI)	*P*-value
Level of E-cadherin expression (high vs. low)	1.423 (0.634–3.192)	0.0455	1.241 (0.553–2.78)	0.0452
Level of Vimentin expression (high vs. low)	1.974(1.147–5.053)	0.0434	2.161(1.178–5.334)	0.0317
Level of Twist expression (high vs. low)	2.350(1.316–5.981)	0.0164	2.248 (1.175–5.327)	0.0227

*HR* hazard ratio, *CI* confidence interval, *DFS* disease-free survival, *OS* overall survival

Higher E-cadherin, Vimentin and Twist expression were independently associated with the poor prognosis of patients with ovarian cancer

### TRPM7 silencing inhibits the invasion and metastasis of ovarian cancer

**Fig. 2:**
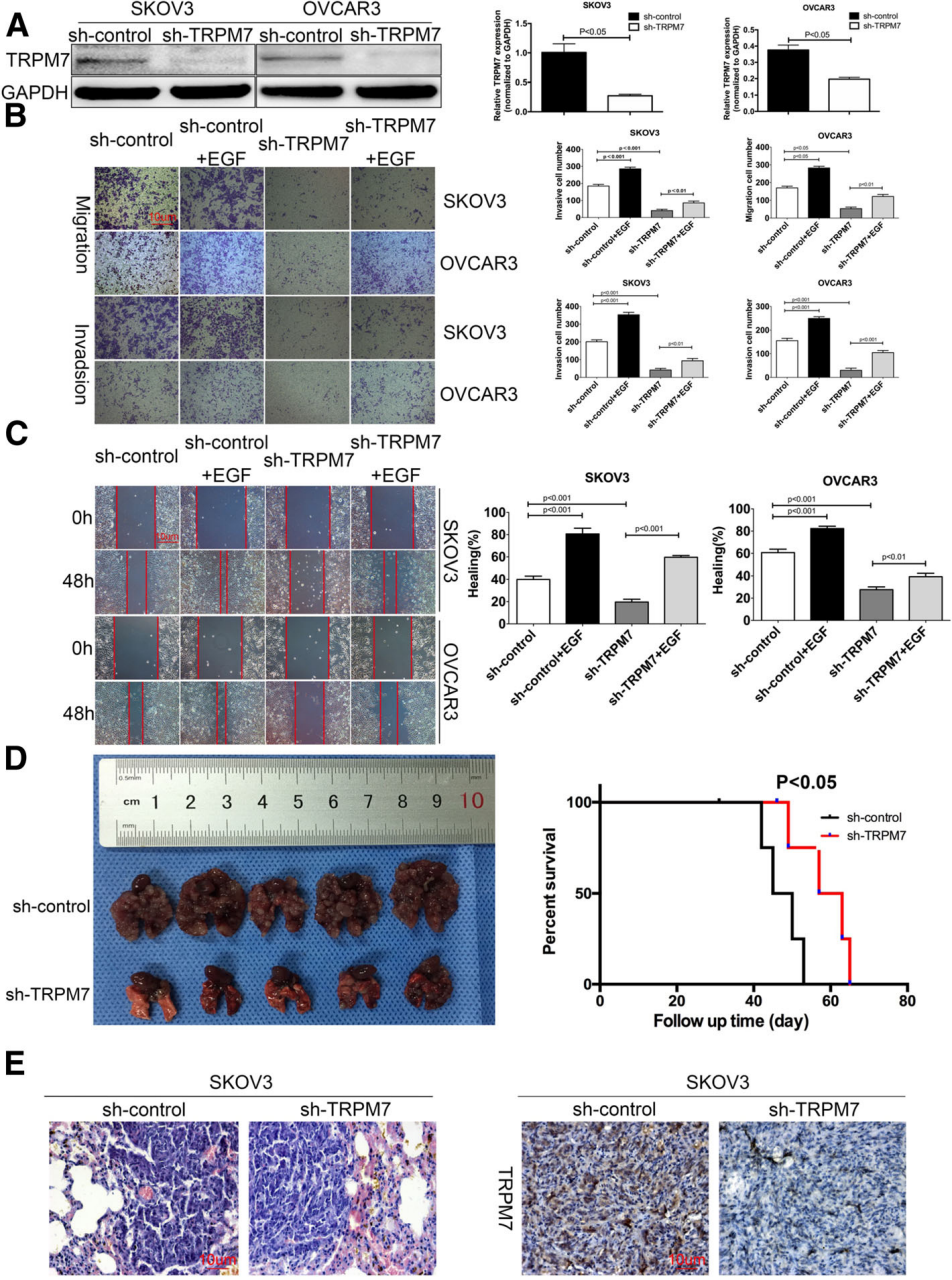
TRPM7 silencing inhibit the migration, invasion and wound healing of ovarian cancer cells and the metastasis of ovarian cancer in mice. SKOV3 and OVCAR3 cells were transfected with plasmid for scrambled RNA or TRPM7-specific shRNA expression to establish SKOV3-sh, SKOV3-TRPM7-sh, OVCAR3-sh and OVCAR3-TRPM7-sh cells. (**a**) Western blot and qRT-PCR analyses of TRPM7 expression. (**b**-**c**) The EGF-induced migration, invasion and wound healing of SKOV3-sh, SKOV3-TRPM7-sh, OVCAR3-sh and OVCAR3-TRPM7-sh cells were determined by transwell migration and invasion and wound healing assays. (**d**) TRPM7 silencing decreases the growth of ovarian cancer and promotes the survival of mice bearing ovarian cancer. BALB/c nude mice were randomized and injected intravenously with SKOV3-sh or SKOV3-TRPM7-sh cells. At 30th post inoculation, the lung tissues were dissected from each group (*n* = 5) of mice and imaged. The remaining mice were monitored for their death (n = 5 per group). (**e**) The lung metastatic ovarian tumors were histologically examined and the expression of TRPM7 in the tumor tissues was determined by immunohistochemistry. Data are representative images or expressed as the mean ± SD of each group from at least three separate experiments

To explore the role of TRPM7 in the development and progression of ovarian cancer, SKOV3 and OVCAR3 cells were transfected with plasmid for control shRNA or TRPM7-sepcific shRNA that displayed the best effect on reducing TRPM7 expression in our experimental system (Fig. 2 and Additional file 2 Figure S1A). Following transfection, SKOV3-Con-sh, SKOV3-TRPM7-sh, OVCAR3-Con-sh and OVCAR3-TRPM7-sh cells were generated, respectively (Fig. 2a). The relative levels of TRPM7 expression in SKOV3-TRPM7-sh and OVCAR3-TRPM7-sh cells were dramatically reduced by 80–60% (*P* < 0.05 for both, Fig. 2a). TRPM7 silencing significantly decreased the migration, invasion and wound healing as well as the EGF-stimulated migration, invasion and wound healing in both SKOV3 and OVCAR3 cells (Fig. 2b and c). Similarly, treatment with MK886 [21–23], a potent 5-lipoxygenase inhibitor, also decreased the levels of TRPM7 expression and reduced the migration, invasion and wound healing as well as EGF-stimulated migration, invasion and wound healing in SKOV3 and OVCAR3 cells (Additional file 2 Figure S1). Furthermore, TRPM7 silencing reduced the numbers and sizes of metastatic lung tumors at 30 days post inoculation and prolonged the survival of tumor-bearing mice (P < 0.05, Fig. 2d). IHC analysis indicated that TRPM7 expression in TRPM7 silenced tumors was obviously lower than that in the control group (Fig. 2e). Together, TRPM7 silencing inhibited the migration, invasion, wound healing of ovarian cancer cells in vitro and lung metastasis in mice.

### TRPM7 silencing attenuates the EMT process of ovarian cancer cells

**Fig. 3:**
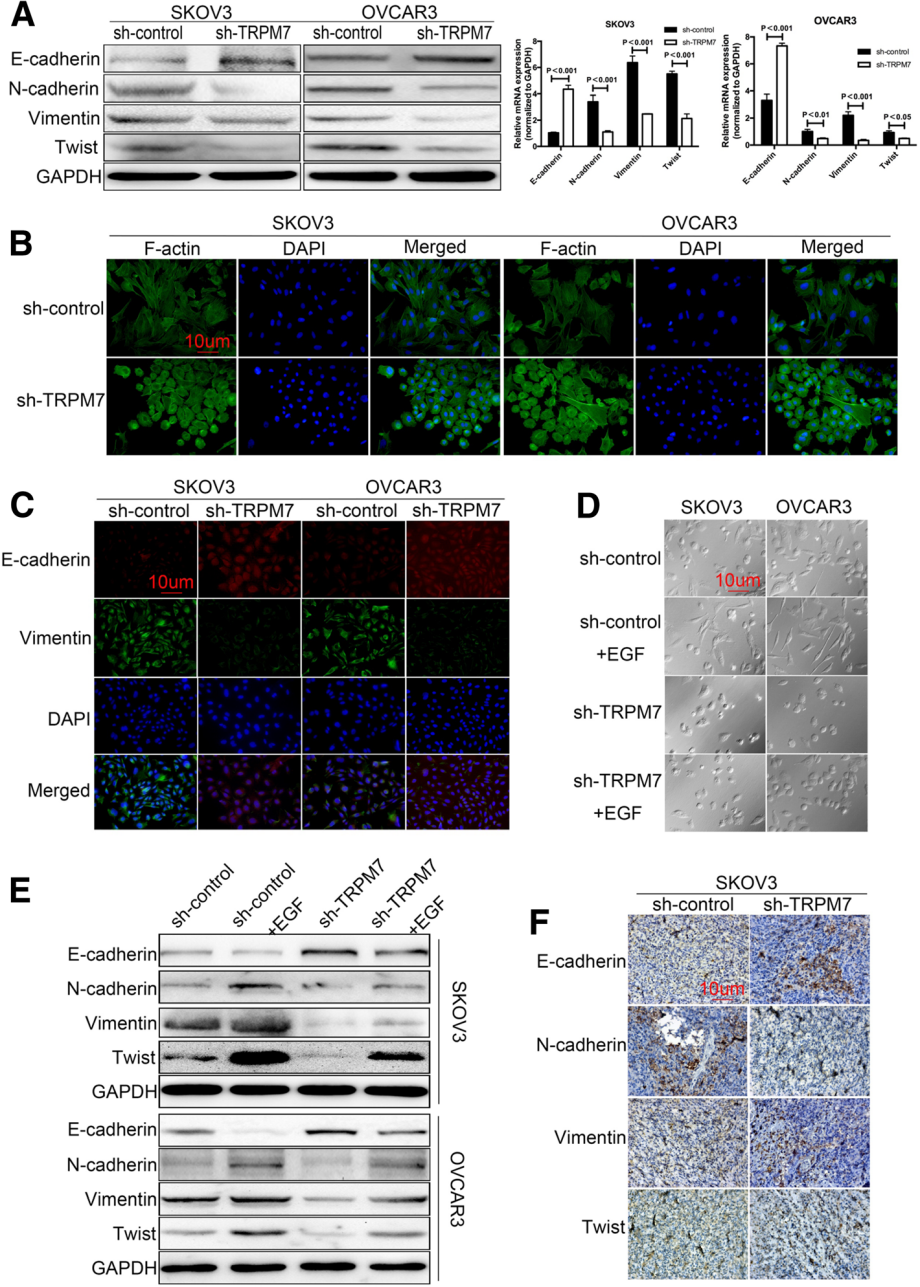
TRPM7 silencing inhibits the EMT in ovarian cancer cells. (**a**) The relative levels of EMT molecule expression in SKOV3-sh, SKOV3-TRPM7-sh, OVCAR3-sh and OVCAR3-TRPM7-sh cells were determined by Western blot and qRT-PCR. (**b**) The levels of F-actin expression in those cells were determined by fluorescent microscopy after staining with Phalloidin-iFluor 488. (**c**) Immunofluorescent analysis of E-cadherin and Vimentin expression in ovarian cancer cells after stained with rabbit anti-E-cadherin and mouse anti-Vimentin and subsequent Alexa Fluor™488-conjugated goat anti-mouse IgG and Alexa Fluor™ 594-conjugated goat anti-rabbit IgG as well as DAPI. (**d**) The cellular morphology of SKOV3-sh, SKOV3-TRPM7-sh, OVCAR3-sh and OVCAR3-TRPM7-sh cells. (**e**) Western blot analysis of the relative levels of EGF-induced E-cadherin, N-cadherin, Vimentin, Twist expression in those cells. (**f**) The levels of E-cadherin, N-cadherin, Vimentin, Twist expression in the lung metastatic ovarian cancer tissues were determined immunohistochemistry. Data are representative images or expressed as the mean ± SD of each group from three separate experiments (magnification × 400, scale bars 20 μm). *p < 0.05, **p < 0.01, ***p < 0.001 vs. the controls

To understand the mechanisms underlying the action of TRPM7 silencing, the relative levels of E-cadherin, N-cadherin, Vimentin and Twist expression in different groups of ovarian cancer cells were determined by Western blot (Fig. 3a). TRPM7 silencing significantly increased the levels of E-cadherin, but decreased the levels of N-cadherin, Vimentin and Twist expression in SKPV3 and OVCAR3 cells. Immunofluorescent assays revealed that TRPM7 silencing reduced the levels of F-actin and Vimentin expression, but enhanced E-cadherin expression in both types of cells (Fig. 3b and c). In addition, treatment with EGF promoted the morphological changes to form spindle-shaped mesenchymal cells in control SKPV3 and OVCAR3 cells, but not TRPM7 silencing cells (Fig. 3d). Similarly, TRPM7 silencing also mitigated the EGF-decreased E-cadherin expression, and the EGF-increased N-cadherin, Vimentin and Twist expression in both types of cells (Fig. 3e). Similar patterns of EMT-related molecule expression and F-actin expression were detected in MK886-treated SKOV3 and OVCAR3 cells (Additional file 3 Figure S2). TRPM7 silencing increased the levels of E-cadherin, but decreased the levels of N-cadherin, Vimentin and Twist expression in tumor tissues (Fig. 3f). Thus, TRPM7 silencing inhibited the EMT process, contributing to its metastatic inhibition in ovarian cancer.

### TRPM7 silencing decreases the levels of intracellular calcium in ovarian cancer cells

**Fig. 4:**
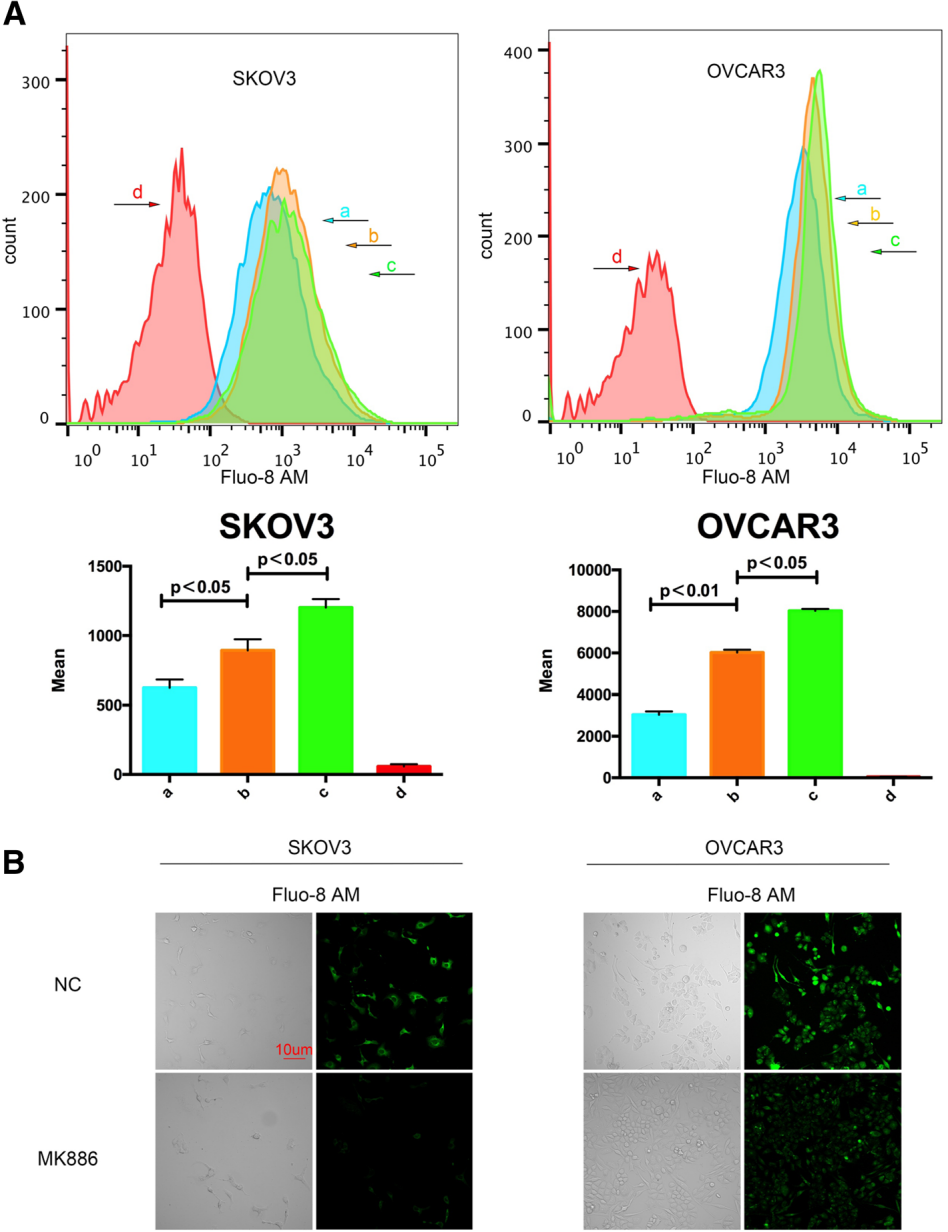
TRPM7 silencing reduces the intracellular calcium concentrations in ovarian cancer cells. (**a**)The fluorescence curves of the SKOV3 and OVCAR3 cells. ‘a’, cells that did not receive any treatment, and were exposed to Ca2 + −free HANK’s solution after uploaded the Fluo-8 AM fluorescence kit. The fluorescence curve of those cells is used as baseline for [Ca2+]I; ‘b’, the cells were treated with MK886 for 24 h, and exposed to HBSS (with CaCl2) after using the Fluo-3 AM fluorescence kit; ‘c’, the cells did not receive any treatment, but were exposed to HBSS (with CaCl2) after using the Fluo-8 AM fluorescence kit; ‘d’, the cells did not receive any treatment and after using the Fluo-8 AM fluorescence kit, the fluorescence curve of those cells was used as a negative control. Following exposure to HBSS (with CaCl2), the fluorescence curve of [Ca2+]I of the cells that TRPM7 silencing was higher than baseline, but lower than the cells that did not receive treatment. This indicated that TRPM7 silencing led to a significant decrease in the [Ca2+]I of the SKOV3 and OVCAR3 cells. (**b**) Fluorescent imaging the [Ca2+]i. SKOV3 and OVCAR3 cells were labeled with Fluo-8 AM and examined under a fluorescent microscope. Data are representative images or expressed as the mean ± SD of each group from three separate experiments

TRPM7 is a cation channel and regulates the calcium-related signaling and cell migration [24]. To understand the molecular mechanisms underlying the action of TRPM7, the impact of TRPM7 on the levels of [Ca^2+^]i was determined by flow cytometry following Fluo-8 AM staining. Treatment with MK886 or TRPM7 silencing decreased the Ca2 + −related fluorescent intensity in SKOV3 and OVCAR3 cells (Fig. 4a). Similarly, fluorescent microscopy indicated that treatment with MK886 reduced the Ca2 + −related fluorescent signals in SKOV3 and OVCAR3 cells (Fig. 4b). Hence, TRPM7 silencing reduced the levels of [Ca^2+^]i in ovarian cancer cells.

### The intracellular calcium is critical for the PI3K / AKT signaling and the migration, invasion and wound healing of ovarian cancer cells

**Fig. 5:**
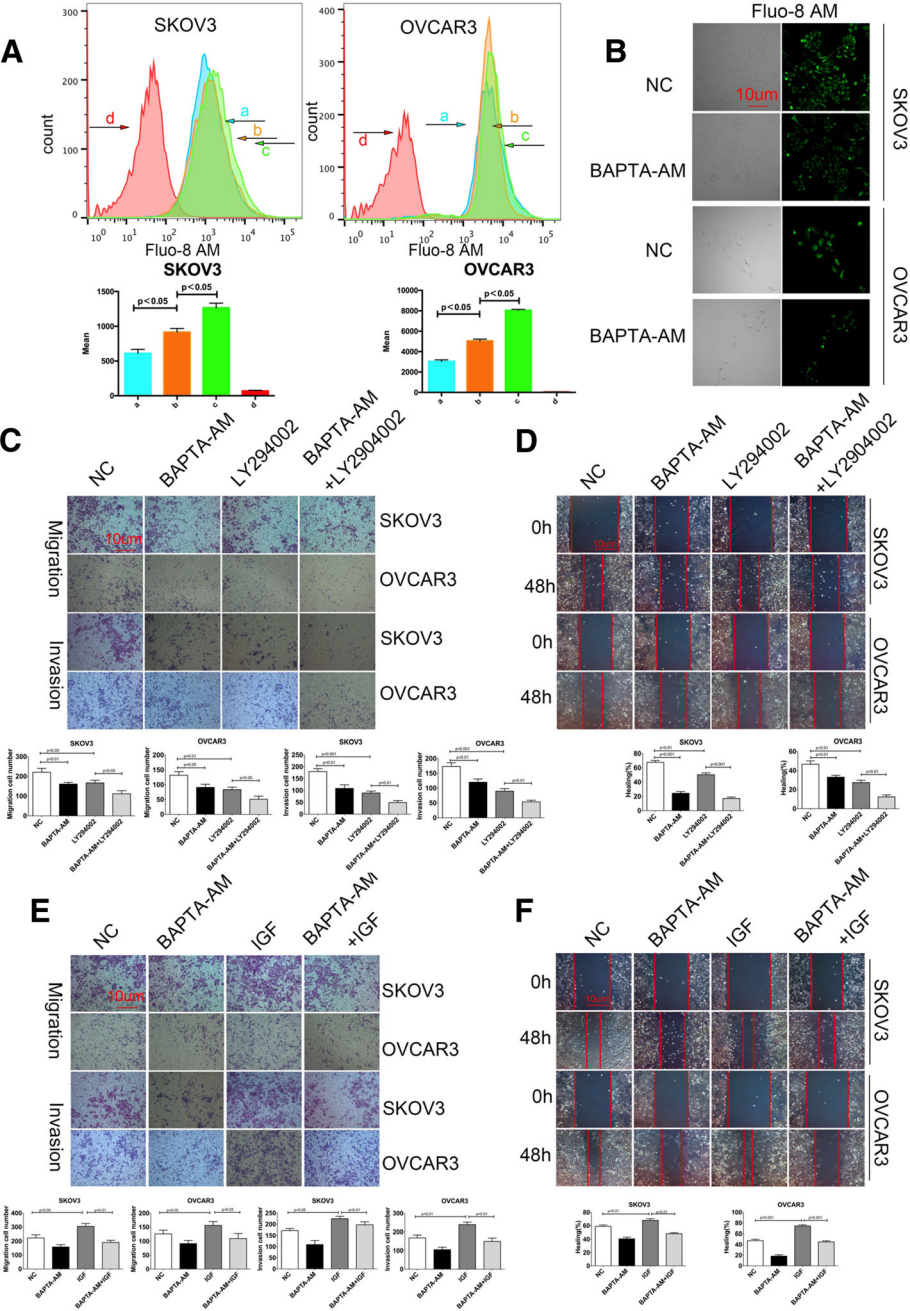
Calcium is crucial for the PI3K/AKT signaling-mediated migration and invasion of ovarian cancer cells. (**a**) Flow cytometry analysis of [Ca2+]i. SKOV3 and OVCAR3 cells were treated with (**b**), or without (**a**), 20 μg/ml BAPTA-AM for 12 h and labeled with Fluo-8 AM, followed by exposed to calcium-containing or calcium-free HANK’s solution, respectively. The cells did not receive BAPTA-AM treatment and exposed to calcium-containing HANK’s solution (**c**); the control cells as described above (**d**). (**b**) Fluorescent microscopy. SKOV3 and OVCAR3 cells were treated with, or without, 20 μg/ml BAPTA-AM for 12 h and labeled with Fluo-8 AM, followed by examining under a fluorescent microscope. (**c**-**f**) Blocking the calcium signaling is crucial for the PI3K/AKT signaling-mediated migration and invasion of ovarian cancer cells. SKOV3 and OVCAR3 cells were treated with vehicle or BAPTA-AM and/or 10 μg/ml LY294002 or 100 ng/ml IGF for 48 h. The migration, invasion (**c**, **e**) and wound healing (**d**, **f**) of cells were determined. Data are representative images or expressed as the mean ± SD of each group of cells from three separate experiments. *p < 0.05, **p < 0.01, ***p < 0.001 vs the controls

The levels of [Ca^2+^]i are crucial for the PI3K/AKT signaling, which regulates the migration and invasion of cancer cells [25, 26]. To explore the potential mechanisms underlying the action of TRPM7 silencing in regulating the migration and invasion of ovarian cancer cells, SKOV3 and OVCAR3 cells were treated with BAPTA-AM, an intracellular calcium chelator for 12 h and the levels of [Ca^2+^]i were determined by Fluo-8 AM staining. In comparison with that of un-treated cells, treatment with BAPTA-AM reduced the levels of [Ca^2+^]i in both SKOV3 and OVCAR3 cells (Fig. 5a). Similarly, treatment with BAPTA-AM decreased the intensity of fluorescent signals in both types of cells (Fig. 5Bb). Functionally, treatment with BAPTA-AM or LY294002, an inhibitor of PI3K, significantly inhibited the migration, invasion and wound healing of SKOV3 and OVCAR3 cells and treatment with both BAPTA-AM and LY294002 further significantly enhanced their inhibitory effects (Fig. 5c and d). It is well known that IGF can activate the PI3K/AKT signaling [27]. We further tested the impact of intracellular calcium depletion on the IGF-induced migration, invasion and wound healing. While treatment with IGF promoted the migration, invasion and wound healing of ovarian cancer cells treatment with BAPTA-AM significantly mitigated the IGF-induced migration, invasion and wound healing in both SKOV3 and OVCAR3 cells in vitro (Fig. 5e and f). Such data indicated that treatment with BAPTA-AM, like TRPM7 silencing, to reduce the levels of [Ca^2+^]i inhibited the migration, invasion and wound healing in ovarian cancer cells by attenuating the PI3K/AKT signaling.

### TRPM7 promotes the invasion and metastasis of ovarian cancer through calcium signal

**Fig. 6:**
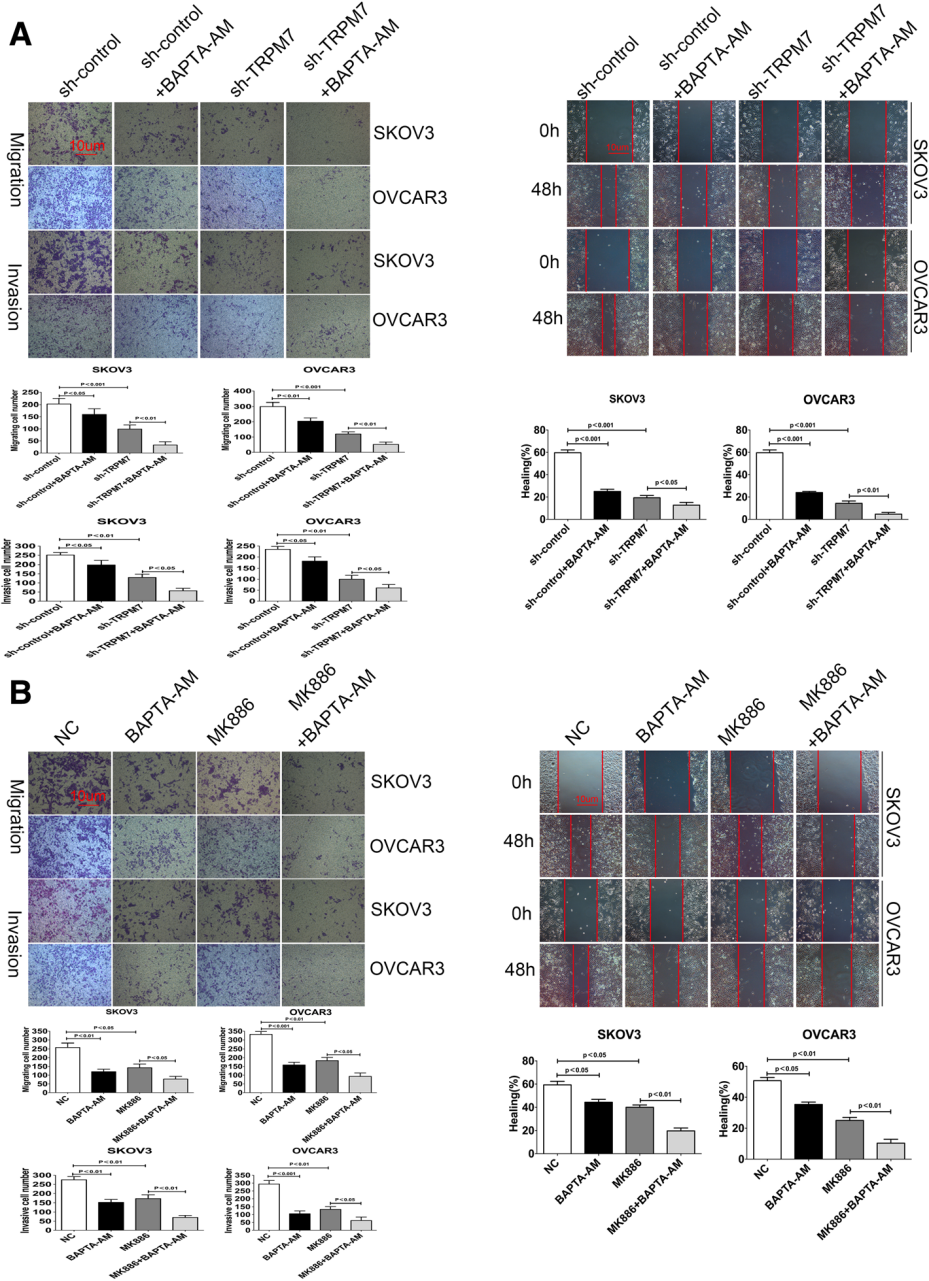
Inhibition of TRPM7 expression enhances the calcium-signaling inhibitor-mediated inhibition of migration, invasion and wound healing in ovarian cancer cells. (**a**) The migration, invasion and wound healing of SKOV3-sh, SKOV3-TRPM7-sh, OVCAR3-sh and OVCAR3-TRPM7-sh cells in the presence or absence of BAPTA-AM were tested for 24 or 48 h. (**b**) Similarly, the migration, invasion and wound healing of SKOV3 and OVCA3 cells in the presence or absence of 30 µg/ml MK886 and/or BAPTA-AM were tested for 24 or 48 h. Data are representative images or expressed as the mean ± SD of each group of cells from three separate experiments

Given that TRPM7 silencing reduced the levels of [Ca^2+^]i and treatment with MK886 inhibited TRPM7 expression we tested the impact of treatment with BAPTA-AM on the migration, invasion and wound healing in the TRPM7 silenced ovarian cancer cells. We found that while treatment with BAPTA-AM significantly reduced the migration, invasion and wound healing in SKOV3-Con-sh and OVCAR3-Con-sh cells and further significantly increased its inhibitory effects in the TRPM7-silenced SKOV3 and OVCAR3 cells (Fig. 6a and b). In paralleling, treatment with both BAPTA-AM and MK886 dramatically reduced the migration, invasion and wound healing in SKOV3 and OVCAR3 cells (Fig. 6c and d). Such data demonstrated that TRPM7 silencing inhibited the migration, invasion and wound healing of ovarian cancer cells by minimizing intracellular free calcium.

### TRPM7 silencing inhibits the EMT process in ovarian cancer cells by attenuating the calcium-related PI3K / AKT signaling

**Fig. 7:**
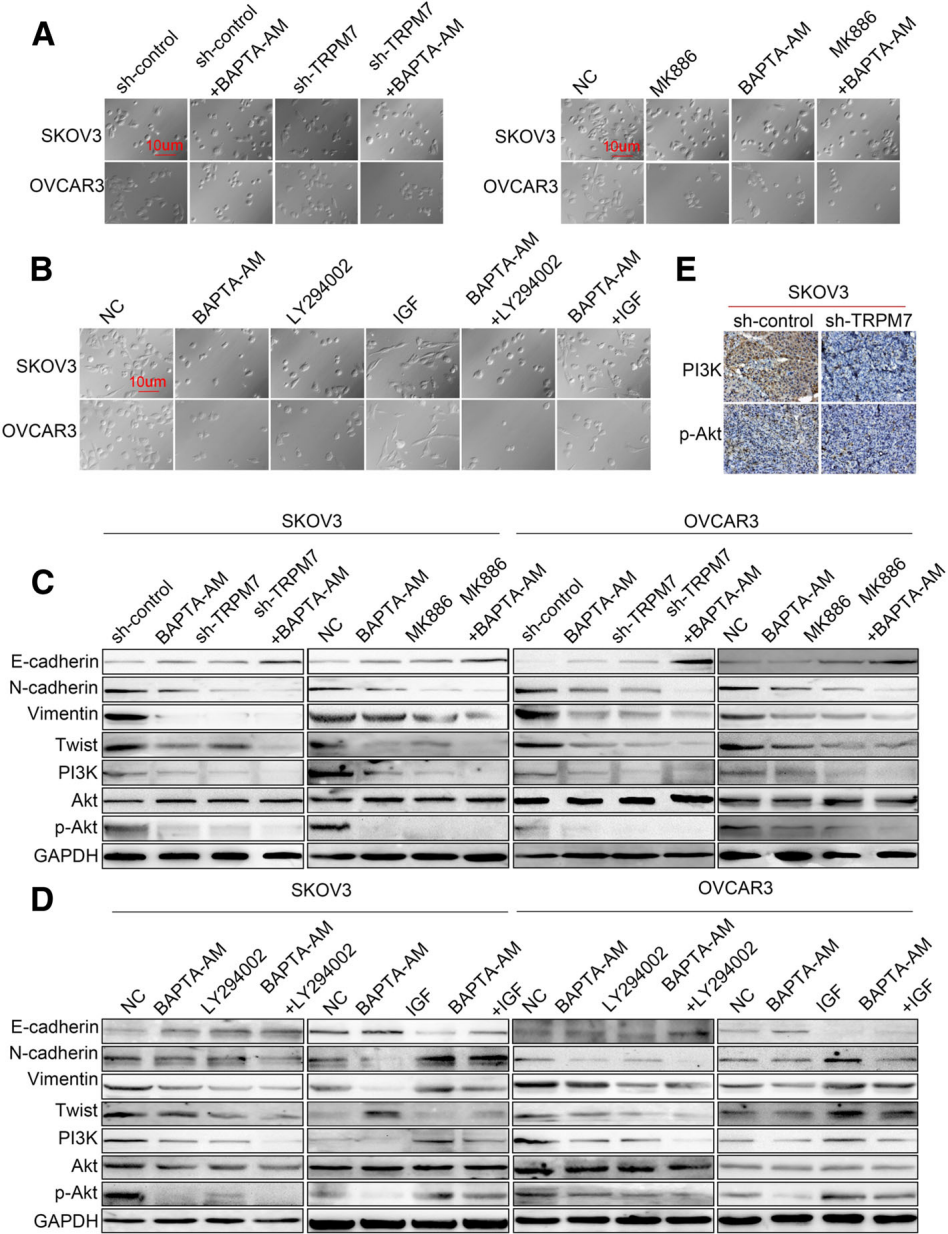
TRPM7 silencing inhibits the EMT by attenuating the calcium -mediated PI3K / AKT signaling in ovarian cancer cells. (**a**) Inhibition of TRPM7 expression alters the morphology of ovarian cancer cells. SKOV3-sh, SKOV3-TRPM7-sh, OVCAR3-sh and OVCAR3-TRPM7-sh cells were treated with vehicle or BAPTA-AM for 24 h. Similarly, SKOV3 and OVCAR3 cells were treated with vehicle or MK886 and/or BAPTA-AM for 24 h. The cell morphology was observed under a light microscope. (**b**) Inhibition of TRPM7 expression modulates the PI3K/AKT inhibitor or IGF-induced morphology in ovarian cancer cells. SKOV3 and OVCAR3 cells were treated with vehicle, BAPTA-AM, and/or LY294002 or IGF for 24 h and the cell morphology was observed. (**c**, **d**) Western blot analysis of the EMT-related molecule expression in individual groups of cells. (**e**) Immunohistochemistry analysis of PI3K expression and AKT phosphorylation in ovarian cancer tissues. Data are representative images of each group of cells from three separate experiments

Finally, we tested whether TRPM7 silencing-decreased EMT process could be attributed to decreased levels of calcium-related PI3K/AKT signaling in ovarian cancer cells. We found that treatment with BAPTA-AM prevented the TRPM7 silencing-induced morphological changes and both SKOV3-TRPM7-sh and OVCAR3-TRPM7-sh cells remained epithelial cobblestone-like morphology (Fig. 7a). Treatment with IGF, but not BAPTA-AM or LY294002, promoted the morphological changes to form spindle-shaped mesenchymal cells in control SKOV3 and OVCAR3 cells, which were mitigated by BAPTA-AM treatment (Fig. 7b). Furthermore, treatment with BAPTA-AM increased the levels of E-cadherin, but decreased the levels of N-cadherin, Vimentin, Twist, PI3K expression and AKT phosphorylation in both SKOV3-Con-sh and SKOV3-TRPM7-sh cells (Fig. 7c). A similar pattern of their expression was detected in the different groups of OVCAR3 cells. In addition, treatment with either BAPTA-AM or LY294002 increased the levels of E-cadherin expression, but decreased the levels of N-cadherin, Vimentin, Twist and PI3K expression and AKT phosphorylation, and treatment with both BAPTA-AM and LY294002 further inhibited the EMT process in SKOV3 and OVCAR3 cells (Fig. 7d). Moreover, treatment with BAPTA-AM also mitigated the IGF-induced EMT process in both SKOV3 and OVCAR3 cells. Finally, there were obviously reduced levels of PI3K expression and AKT phosphorylation in the TRPM7-silenced SKOV3 tumors, compared with that in the SKOV3 tumors (Fig. 7e). Collectively, such data demonstrated that TRPM7 silencing inhibited the EMT process in ovarian cancer cells by attenuating the calcium-related PI3K/AKT signaling.

## Discussion

The metastasis of ovarian cancer is associated with poor prognosis of ovarian cancer patients [28, 29]. TRPM7 is involved in various physiological and pathological processes, including cell proliferation, cycling progression, migration, and remodeling of cells [30–32]. Our previous studies have suggested that TRPM7 may be a prognostic factor for patients with ovarian cancer [14, 20]. In this study, we found that up-regulating TRPM7 expression was correlated with increased EMT process in ovarian cancer tissues and with poor disease-free and overall survival of ovarian cancer patients in this population. Such data were extended our previous observations [14, 20]. Given that the high levels of EMT process are associated with metastasis our findings suggest that up-regulated TRPM7 expression may a valuable factor to predict the metastasis of ovarian cancer [33–35].

Previous studies have shown that EGF is crucial for the migration, invasion and wound healing [36–38] and both F-actin and Vinculin regulate the cytoskeletal remodeling and cell-cell adhesion, to promote cell migration movement [39, 40]. In this study, we found that TRPM7 silencing by specific shRNA significantly decreased the levels of TRPM7 expression and attenuated the EGF-induced migration, invasion and wound healing of ovarian cancer cells in vitro. TRPM7 silencing inhibited the lung metastasis of implanted ovarian cancer in mice and prolonged the survival of tumor-bearing mice. TRPM7 silencing enhanced the EMT and F-actin expression and mitigated the EGF-enhanced EMT process in ovarian cancer cells. Furthermore, inhibition of TRPM7 expression by MK886 also significantly attenuated the EGF-induced migration, invasion and wound healing of ovarian cancer cells by inhibiting the EMT process. Such data support the notion that higher levels of TRPM7 expression are associated with the metastasis of ovarian carcinoma [36–38]. Hence, TRPM7 may be new therapeutic target for prevention and intervention of ovarian cancer metastasis.

The mechanisms by which the calcium signaling regulates tumor EMT process have not been clarified [41–43]. TRPM7 acts as a calcium channel and is crucial for the levels of [Ca^2+^]i in cells [44, 45]. We found that TRPM7 silencing dramatically reduced the levels of TRPM7 expression and the levels of [Ca^2+^]i in ovarian cancer cells. Similarly, treatment with BAPTA-AM to inhibit the calcium channel also reduced the levels of [Ca^2+^]i, consistent with previous observations [43, 46, 47] and attenuated the IGF-induced migration, invasion and wound healing in ovarian cancer cells. Accordingly, such data indicate that both the calcium signaling and the PI3K/AKT pathway positively promote the migration and invasion of ovarian cancer cells [48, 49]. It is possible that the calcium signaling is an important regulator of the PI3K/AKT activation, which promotes the EMT process and metastasis of ovarian cancer [25, 50]. Actually, we found that BAPTA-AM further enhanced the PI3K inhibitor- or TRPM7 silencing-decreased migration, invasion and wound healing in ovarian cancer cells. Similarly, BAPTA-AM also further deteriorated the MK886-decreased migration and invasion of ovarian cancer cells. Thus, TRPM7 silencing decreased the calcium signaling and inhibited the PI3K/AKT activation to attenuate the EMT process, impairing migration and invasion of ovarian cancer cells. Therefore, our findings may provide new insights in the mechanisms underlying the regulation of TRMP7 on the migration, invasion and metastasis of ovarian cancer cells.

In summary, up-regulated TRPM7 expression was correlated with high levels of EMT process in ovarian cancer and associated with shorter survival of patients with ovarian cancer in this population. TRPM7 silencing, inhibition of TRPM7 expression or inhibition of the calcium channel decreased the levels of [Ca^2+^]I and attenuated the EMT process, migration, invasion and wound healing of ovarian cancer cells by inhibiting the PI3K/AKT activation. Thus, TRPM7 levels may be valuable for the prognosis of metastasis and TRPM7 may be a therapeutic target for prevention and intervention of metastasis of ovarian cancer.

## Conclusions

TRPM7 silencing inhibited the EMT and metastasis of ovarian cancer by attenuating the calcium-related PI3k/AKT activation. Our findings suggest that TRPM7 may be a therapeutic target for intervention of ovarian cancer.

## Additional files

Additional file 1: **Table 2.** Detailed information of the patients (*n* = 80). (DOCX 18 kb)

Additional file 2: **Figure S1.** Inhibition of TRPM7 expression by MK886 inhibits the migration, invasion and wound healing of ovarian cancer cells. SKOV3 and OVCAR3 cells were transduced with lentiviral for expressing scramble sequence, TRPM7-sh1, TRPM7-sh2, or TRPM7-sh3, respectively, for 4 days and the relative levels of TRPM7 expression were determined by Western blotting (**A**). SKOV3 and OVCAR3 cells were treated with vehicle (NC) or 30 μg/ml MK886 for 48 h. The relative levels of TRPM7 mRNA transcripts and protein expression were determined by quantitative RT-PCR and Western blot (**B**). (**C**, **D**) Inhibition of TRPM7 expression by MK886 mitigated the EGF-induced migration, invasion and wound healing of ovarian cancer cells. The migration, invasion and wound healing of SKOV3 and OVCAR3 cells were tested in the presence or absence of EGF and/or MK886 for 48 h. Data are representative images or expressed as the mean ± SD of each group of cells from three separate experiments. (PDF 2592 kb)

Additional file 3: **Figure S2.** Inhibition of TRPM7 expression by MK886 inhibits the EMT process of ovarian cancer cells. SKOV3 and OVCAR3 cells were treated with vehicle (NC) or 30 μg/ml MK886 for 48 h. (**A**) Western blot analysis of the relative levels of E-cadherin, N-cadherin, Vimentin, and Twist to GAPDH. (**B**) Fluorescent microscopy analysis of F-actin expression. (**C**) Immunofluorescent analysis of E-cadherin and Vimentin expression. SKOV3 and OVCAR3 cells were treated with vehicle (NC) or EGF in the presence or absence of MK886 for 48 h. The morphology (**D**) and the relative levels of E-cadherin, N-cadherin, Vimentin, Twist expression (**E**) were determined by microscopy and Western blot assays, respectively. Data are representative images or expressed as the mean ± SD of each group of cells from three separate experiments. *p < 0.05, **p < 0.01, ***p < 0.001 vs the controls. (PDF 1573 kb)

## Abbreviations

CI: Confidence interval
DFS: Disease-free survival
EGF: Epidermal growth factor
EMT: Epithelial-mesenchymal transition
H + E: Hematoxylin and eosin
HBSS: Hank’s balanced salt solution
HR: Hazard ratio
HRP: Horseradish peroxidase
IGF: Insulin-like growth factors
IHC: Immunohistochemistry
MMP: Matrix metalloproteinase
OS: Overall survival
PVDF: Polyvinylidene difluoride
qRT-PCR: Quantitative real-time PCR
SDS-PAGE: Sodium dodecyl sulfate-polyacrylamide gel electrophoresis;
TRPM7: Transient receptor potential 7
α-SMA: α smooth muscle actin
