# Correction: TRPM7 promotes the epithelial– mesenchymal transition in ovarian cancer through the calcium-related PI3K / AKT oncogenic signaling

**DOI:** 10.1186/s13046-024-03212-7

**Published:** 2024-10-21

**Authors:** Lu Liu, Nayiyuan Wu, Ying Wang, Xiaoyun Zhang, Bing Xia, Jie Tang, Jingting Cai, Zitong Zhao, Qianjin Liao, Jing Wang

**Affiliations:** 1 Hunan clinicaI Research Center in Gynecologic Cancer, Hunan Cancer Hospital and The Affiliated Cancer Hospital of Xiangya School of Medicine, Central South University, 283, Tongzipo Road, Changsha, 410013 Hunan People’s Republic of China; 2 University of South China, Hengyang, 421001 People’s Republic of China

**Correction** **:** **J Exp Clin Cancer Res 38, 106 (2019)**

**https://doi.org/10.1186/s13046-019-1061-y**

Following publication of the original article [1], the authors identified minor errors in image typesetting in Fig. 5, specifically the invasion experiment detailed in Fig. 5C

The corrections do not have any effect on the results or conclusions of the paper. The correct figure is presented below:

**Incorrect** Fig. 5

**Fig. 5:**
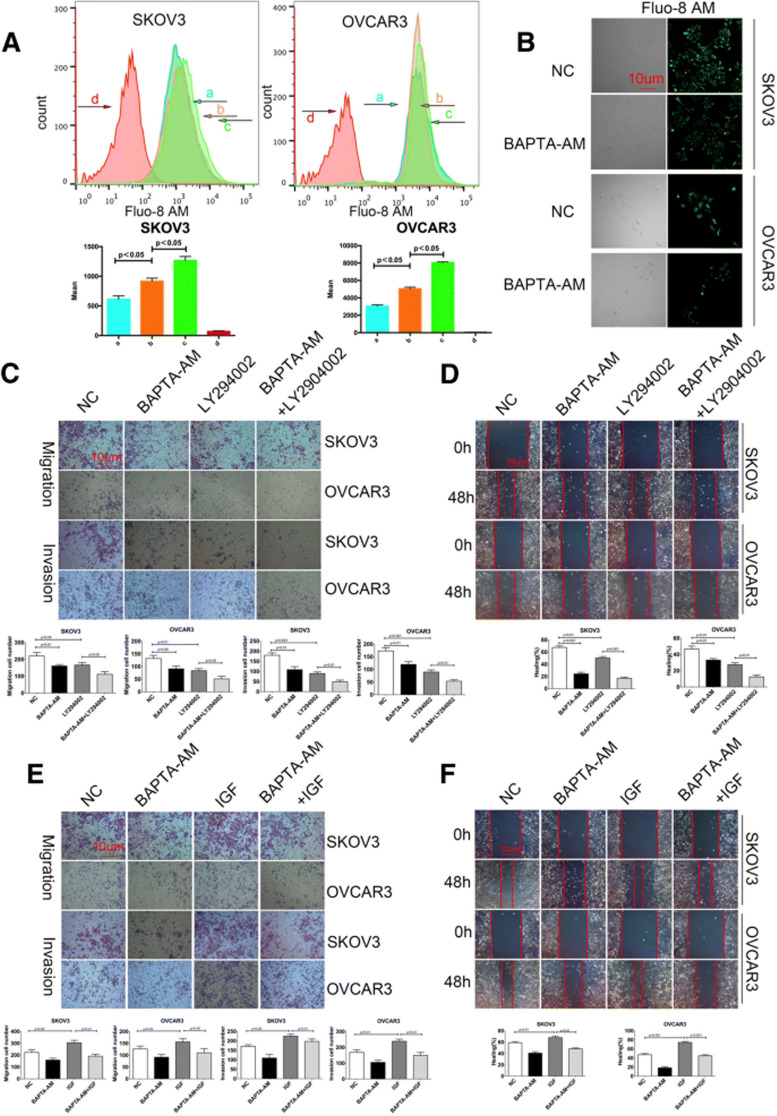
Calcium is crucial for the PI3K/AKT signaling-mediated migration and invasion of ovarian cancer cells. (**a**) Flow cytometry analysis of [Ca2 +]i. SKOV3 and OVCAR3 cells were treated with (**b**), or without (**a**), 20 μg/ml BAPTA-AM for 12 h and labeled with Fluo-8 AM, followed by exposed to calcium-containing or calcium-free HANK’s solution, respectively. The cells did not receive BAPTA-AM treatment and exposed to calciumcontaining HANK’s solution (**c**); the control cells as described above (**d**). (**b**) Fluorescent microscopy. SKOV3 and OVCAR3 cells were treated with, or without, 20 μg/ml BAPTA-AM for 12 h and labeled with Fluo-8 AM, followed by examining under a fluorescent microscope. (**c-f**) Blocking the calcium signaling is crucial for the PI3K/AKT signaling-mediated migration and invasion of ovarian cancer cells. SKOV3 and OVCAR3 cells were treated with vehicle or BAPTA-AM and/or 10 μg/ml LY294002 or 100 ng/ml IGF for 48 h. The migration, invasion (**c, e**) and wound healing (**d, f**) of cells were determined. Data are representative images or expressed as the mean ± SD of each group of cells from three separate experiments. *p < 0.05, **p < 0.01, ***p < 0.001 vs the controls

**Correct **Fig. 5

**Fig. 5:**
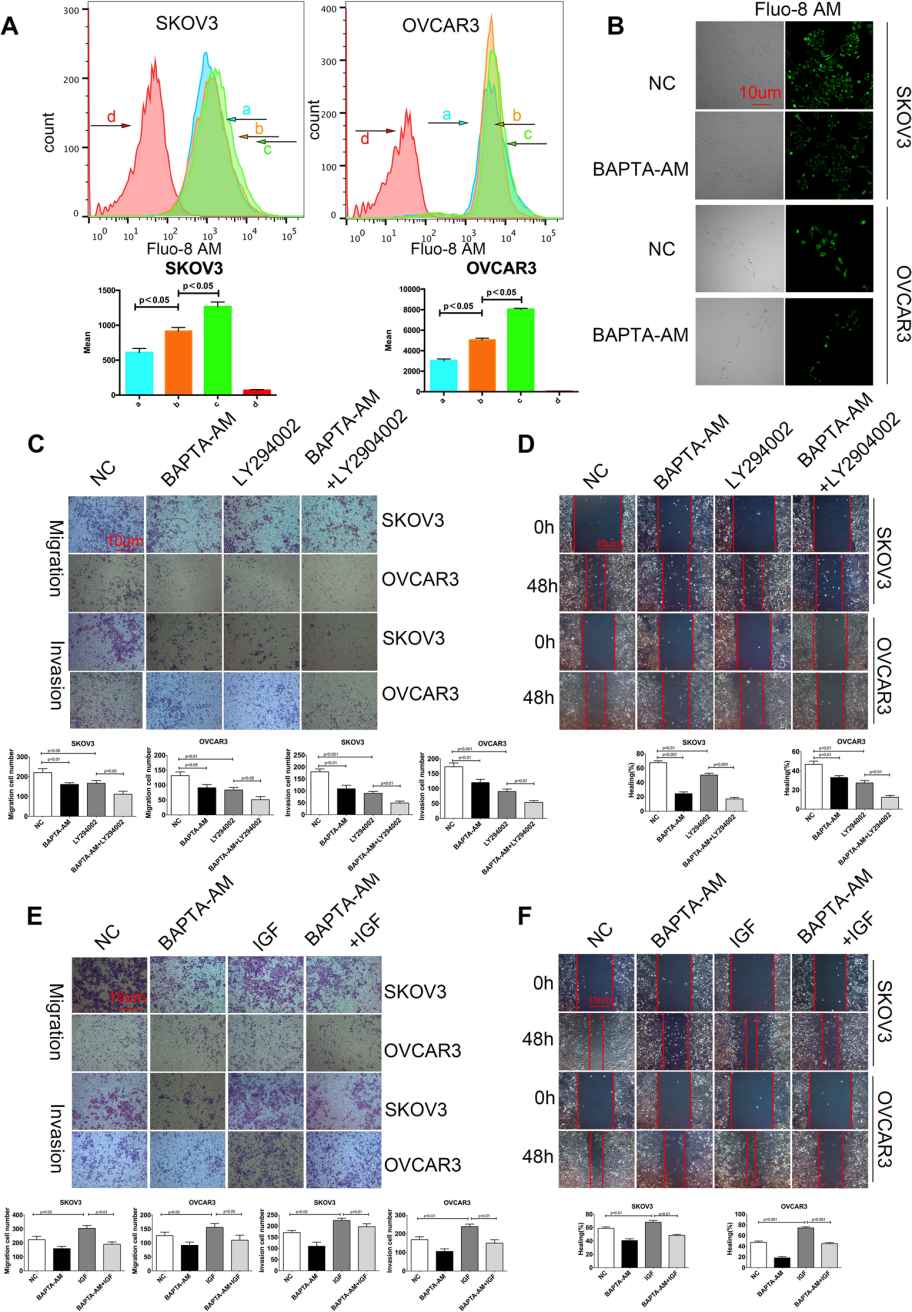
Calcium is crucial for the PI3K/AKT signaling-mediated migration and invasion of ovarian cancer cells. (**a**) Flow cytometry analysis of [Ca2 +]i. SKOV3 and OVCAR3 cells were treated with (**b**), or without (**a**), 20 μg/ml BAPTA-AM for 12 h and labeled with Fluo-8 AM, followed by exposed to calcium-containing or calcium-free HANK’s solution, respectively. The cells did not receive BAPTA-AM treatment and exposed to calciumcontaining HANK’s solution (**c**); the control cells as described above (**d**). (**b**) Fluorescent microscopy. SKOV3 and OVCAR3 cells were treated with, or without, 20 μg/ml BAPTA-AM for 12 h and labeled with Fluo-8 AM, followed by examining under a fluorescent microscope. (**c-f**) Blocking the calcium signaling is crucial for the PI3K/AKT signaling-mediated migration and invasion of ovarian cancer cells. SKOV3 and OVCAR3 cells were treated with vehicle or BAPTA-AM and/or 10 μg/ml LY294002 or 100 ng/ml IGF for 48 h. The migration, invasion (**c, e**) and wound healing (**d, f**) of cells were determined. Data are representative images or expressed as the mean ± SD of each group of cells from three separate experiments. *p < 0.05, **p < 0.01, ***p < 0.001 vs the controls
