# Supplementary material for: TRPM7 promotes the epithelial–mesenchymal transition in ovarian cancer through the calcium-related PI3K / AKT oncogenic signaling
Source: J Exp Clin Cancer Res. 2019 Feb 28;38:106. doi: 10.1186/s13046-019-1061-y (PMC6396458; doi:10.1186/s13046-019-1061-y)
Supplement: Supplementary file 1 — Table 2. Detailed information of the patients (n = 80). (DOCX 18 kb) [file 13046_2019_1061_MOESM1_ESM.docx]

**Table 2. Detailed information of the patients (n=80)**

| Characteristics | No. of patients |
| --- | --- |
| Ages  ≤60  >60 | 37  43 |
| Diagnosis ovarian cancer  non-tumor | 60  20 |
| Histologic type  Serous  Mucinous  Endometrioid  non-tumor | 46  9  5  20 |
| Pathological grade  1  2  3  non-tumor | 11  18  31  20 |
| FIGO stage  I-II  III-IV  non-tumor | 23  37  20 |
| Pelvic metastasis  YES  NO | 46  34 |

Abbreviation, FIGO, International Federation of Gynecology and Obstetrics
