# Supplementary figures and images for: TRPM7 promotes the epithelial–mesenchymal transition in ovarian cancer through the calcium-related PI3K / AKT oncogenic signaling
Source: J Exp Clin Cancer Res. 2019 Feb 28;38:106. doi: 10.1186/s13046-019-1061-y (PMC6396458; doi:10.1186/s13046-019-1061-y)

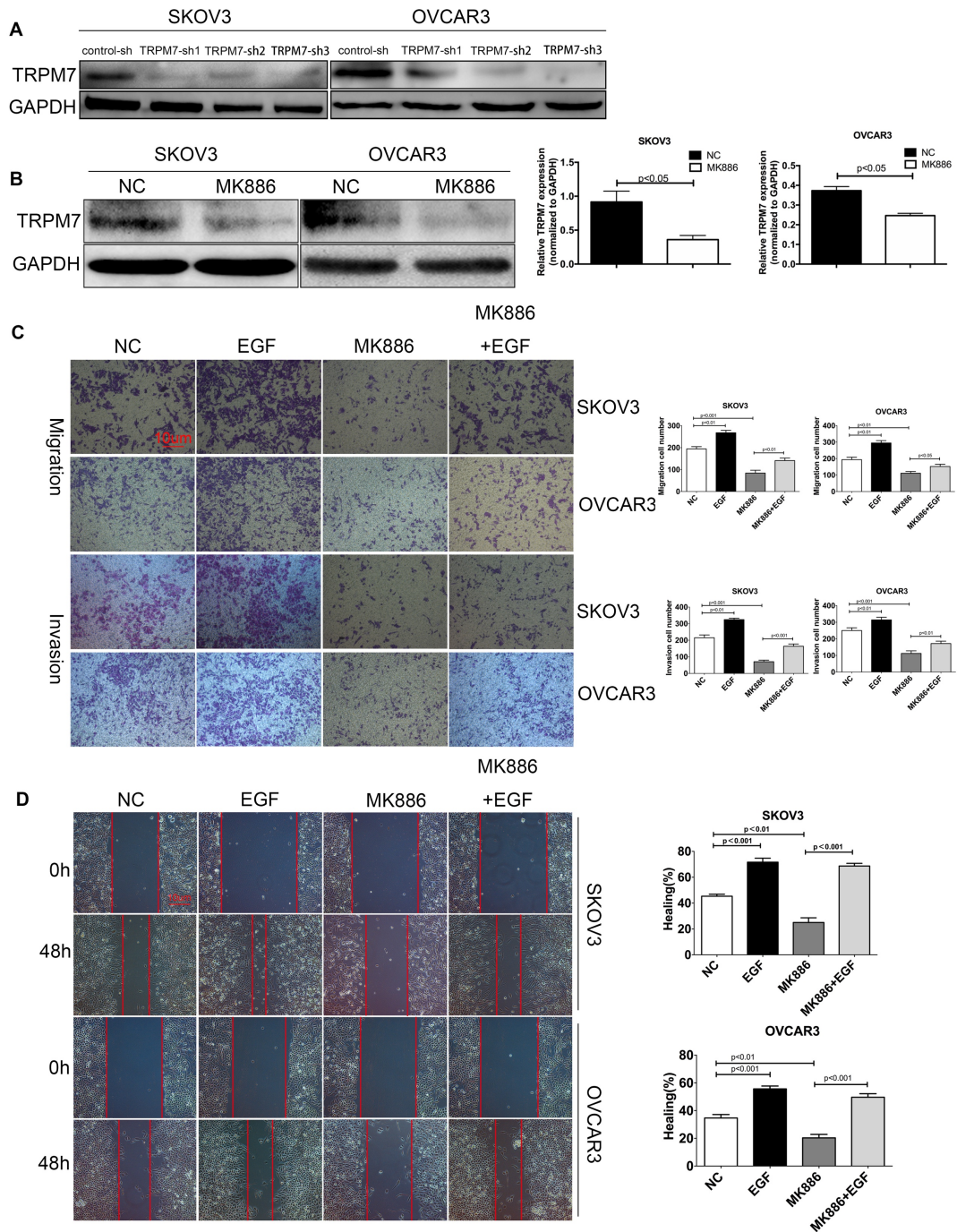

Supplement: Supplementary file 2 — Figure S1. Inhibition of TRPM7 expression by MK886 inhibits the migration, invasion and wound healing of ovarian cancer cells. SKOV3 and OVCAR3 cells were transduced with lentiviral for expressing scramble sequence, TRPM7-sh1, TRPM7-sh2, or TRPM7-sh3, respectively, for 4 days and the relative levels of TRPM7 expression were determined by Western blotting (A). SKOV3 and OVCAR3 cells were treated with vehicle (NC) or 30 μg/ml MK886 for 48 h. The relative levels of TRPM7 mRNA transcripts and protein expression were determined by quantitative RT-PCR and Western blot (B). (C, D) Inhibition of TRPM7 expression by MK886 mitigated the EGF-induced migration, invasion and wound healing of ovarian cancer cells. The migration, invasion and wound healing of SKOV3 and OVCAR3 cells were tested in the presence or absence of EGF and/or MK886 for 48 h. Data are representative images or expressed as the mean ± SD of each group of cells from three separate experiments. (PDF 2592 kb) [file 13046_2019_1061_MOESM2_ESM.pdf]

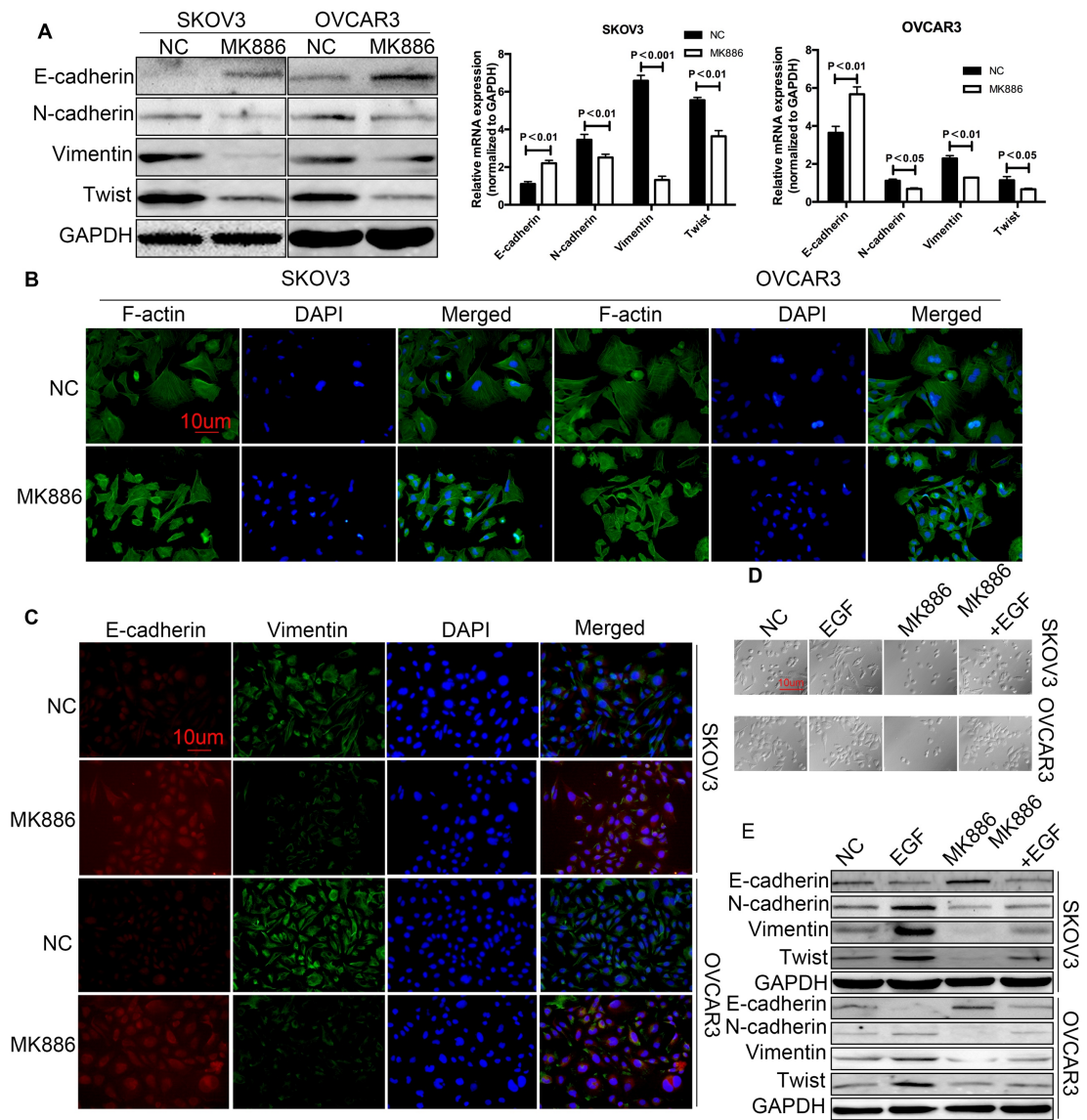

Supplement: Supplementary file 3 — Figure S2. Inhibition of TRPM7 expression by MK886 inhibits the EMT process of ovarian cancer cells. SKOV3 and OVCAR3 cells were treated with vehicle (NC) or 30 μg/ml MK886 for 48 h. (A) Western blot analysis of the relative levels of E-cadherin, N-cadherin, Vimentin, and Twist to GAPDH. (B) Fluorescent microscopy analysis of F-actin expression. (C) Immunofluorescent analysis of E-cadherin and Vimentin expression. SKOV3 and OVCAR3 cells were treated with vehicle (NC) or EGF in the presence or absence of MK886 for 48 h. The morphology (D) and the relative levels of E-cadherin, N-cadherin, Vimentin, Twist expression (E) were determined by microscopy and Western blot assays, respectively. Data are representative images or expressed as the mean ± SD of each group of cells from three separate experiments. *p < 0.05, **p < 0.01, ***p < 0.001 vs the controls. (PDF 1573 kb) [file 13046_2019_1061_MOESM3_ESM.pdf]
